# Dermatochalasis Aggravates Meibomian Gland Dysfunction Related Dry Eyes

**DOI:** 10.3390/jcm11092379

**Published:** 2022-04-23

**Authors:** Wan-Lin Wu, Shu-Wen Chang

**Affiliations:** 1 Department of Ophthalmology, Far Eastern Memorial Hospital, New Taipei City 220, Taiwan; arielwu10101@gmail.com; 2 Department of Ophthalmology, National Taiwan University Hospital, Taipei 100, Taiwan; 3 School of Medicine, National Taiwan University, Taipei 100, Taiwan

**Keywords:** dermatochalasis, dry-eye, tear-film lipid-layer, meibomian gland dysfunction, age, expressible meibomian gland, meiboscale

## Abstract

This study aimed to investigate the relationships between subjective symptoms, objective signs, and dermatochalasis severity in dry-eye patients and the effects of lid hygiene on dry-eye parameters. We retrospectively enrolled 2328 patients who underwent dry-eye examinations and classified them into four groups by dermatochalasis severity. The SPEED and OSDI questionnaires were used to evaluate subjective symptoms. LipiView^®^ II interferometry was used to measure lipid-layer thickness (LLT) and blink/incomplete blink rates and perform meibography. A slit-lamp–aided standardized evaluator measured meibomian gland expressibility (MGE). A meiboscale was used to grade meibomian gland dropout. Fluorescein tear-film break-up time (FTBUT) and superficial punctate keratitis (SPK) were recorded. The Schirmer test II with anesthetics was used to evaluate aqueous tear secretion. The effects of lid hygiene were evaluated in 644 patients who underwent second comprehensive examinations. The median age of patients was 55.3 [46.0–66.0] years (76.0% female). Patients with more severe dermatochalasis were less symptomatic and had less MGE, higher meiboscale grades and average LLT. Dermatochalasis severity was significantly associated with MGE and meiboscale grade in the upper lid. There were no significant differences in the Schirmer test, FTBUT, and SPK among the severity groups. Females were older and had higher LLT and less severe dermatochalasis. Lid hygiene significantly decreased subjective symptoms, LLT, and Schirmer results, increased FTBUT, but did not change MGE or meiboscale grades. Dermatochalasis severity participated in the pathophysiology of dry eyes. Lid hygiene significantly improved subjective symptoms and reduced LLT, more significantly in patients with less severe dermatochalasis.

## 1. Introduction

Dermatochalasis is a common sign of periocular aging often seen in middle-aged and elderly people and is characterized by loose and redundant eyelid skin. Histologically, dermatochalasis is associated with macrophage-related subclinical inflammation, elastolysis, lymphostasis, reduced elastic fibers and disarranged collagen fibers [1,2]. Dermatochalasis-associated lymphangiectasia progresses significantly with age [3]. Moreover, skeletal muscle mass decreases by 3–8% per decade from the age of 30 years and increases further after the age of 65 years [4]. The orbicularis oculi muscle is a skeletal muscle that demonstrates fiber thinning with age [5]. Decreased orbicularis oculi muscle mass contributes to decreased muscle strength and function in the elderly [6]. Blinking is associated with the orbicularis oculi muscle and meibum secretion [7,8], but less so with the overlying eyelid skin. Despite this, dermatochalasis-associated redundant skin can interfere with lid hygiene and cause lid-margin inflammation and meibomian gland dysfunction (MGD). However, the effect of dermatochalasis on meibomian gland function has been less well studied.

The Standardized Patient Evaluation of Eye Dryness (SPEED) questionnaire assesses the frequency and severity of subjective symptoms [9], and the Ocular Surface Disease Index (OSDI) questionnaire assesses the frequency of dry-eye symptoms and their effect on vision-related functions [10]. Although the SPEED and OSDI questionnaire scores are significantly correlated [11], the SPEED questionnaire scores more strongly correlate with evaporative dry-eye parameters, whereas the OSDI questionnaire scores more strongly correlate with aqueous tear-deficient dry-eye parameters [12]. However, age and self-perceived health affect the scoring of subjective symptoms, whereas the presence of systemic diseases, including Sjögren syndrome, facial rosacea, rheumatoid arthritis, and peripheral artery disease as well as smoking history, increases the objective signs [11,13,14,15]. This could contribute to the discordance between the subjective symptoms and objective signs during dry-eye assessment.

Aging leads to changes in the meibomian gland but not in the meibum component [16]. Interferometry is used to measure the lipid-layer thickness (LLT) and blink patterns of each patient. Together with meibomian gland secretion evaluation and meibography, lipid-layer interferometry is a useful technique for dry-eye assessment, both at initial diagnosis and treatment follow-up for MGD [11,14]. The amount of lipid secretion in MGD correlates with dry-eye parameters and ocular symptoms [11,17,18,19]. Meibomian dropout has been widely used as a diagnostic and treatment follow-up parameter for MGD [11,18,20,21,22]. Lid hygiene has been advocated in the management of MGD [23,24]. However, compliance in dry-eye disease management is a major challenge [25], and the effects of lid hygiene on meibomian gland (MG) parameters have been less well delineated. In this study, we evaluated the severity of dermatochalasis and its impact on dry-eye patients. We also evaluated the effect of lid hygiene treatment on subjective and objective dry-eye parameters.

## 2. Materials and Methods

### 2.1. Patients

This study was conducted in accordance with the Declaration of Helsinki according to a protocol approved by the Institutional Review Board at Far Eastern Memorial Hospital. We retrospectively reviewed the electronic medical records of dry-eye patients at Far Eastern Memorial Hospital Dry-Eye Center between November 2015 and December 2020. The included patients were >20 years old. We excluded patients who had (1) previous ocular surgery other than cataract surgery, (2) cataract surgery in the past 6 months, (3) previous ocular trauma or chemical injury, (4) active ocular infection, allergy (including allergic conjunctivitis, atopic keratoconjunctivitis, and vernal keratoconjunctivitis), graft-versus-host disease, Steven–Johnson syndrome, mucous membrane pemphigoid, (5) current topical ophthalmic medications other than artificial tears (including antihistamine, mast-cell stabilizer, antiglaucoma agents, steroid, antibiotics, and (6) oral antihistamine), tetracycline, doxycycline, or minocycline. The patients were included and excluded on the basis of chart review. Those who used artificial tears, gel, or ointment on a regular basis were instructed to stop for ≥12 h before the examination. Contact lens wearers were instructed to stop wearing ≥1 day before examination. Patients were instructed not to wear periocular cosmetics on the day of the examination.

**Figure 1:**
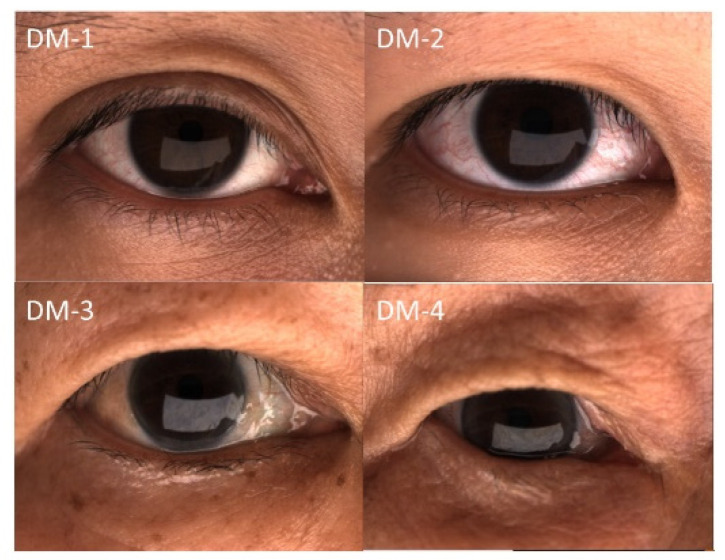
Representative external eye photograph of eyelid configuration.

After dry-eye center examinations, the patients were instructed to conduct lid hygiene by washing their eyelids, especially the dermatochalasis skin folds, with horizontal movement of a finger and the use of facial soap and lukewarm water. A 15-min warm compress followed by 15 forceful blinks twice daily was recommended after the first comprehensive dry-eye assessment. Topical medications, including 0.1% fluorometholone for 1–3 months and artificial tears, were prescribed. Follow-up comprehensive dry-eye evaluations were scheduled at 3 months after the first examination. The patients were divided into four groups according to the degree of dermatochalasis (DM) (Figure 1) [26], including DM-1: normal, the upper-eyelid skin does not touch the eyelashes; DM-2: mild, the upper-eyelid skin just touches the eyelashes; DM-3: moderate, the upper-eyelid skin hangs over the eyelashes; and DM-4: severe, the upper-eyelid skin hangs over the eye. Dermatochalasis severity was evaluated by one well-trained technician. Data from the right eyes were used for analysis to avoid overestimation.

### 2.2. Dry-Eye Examination Protocol

All participants completed a series of examinations in our dry-eye center and completed the SPEED and OSDI questionnaires to quantify subjective symptoms. The two components of SPEED were included for analysis: frequency and severity. Similarly, the three components of OSDI were included for analysis: OSDI (A)—frequency of symptoms; OSDI (B)—frequency of activity limitation; OSDI (C)—frequency of environmental factors triggered discomfort.

**Figure 2:**
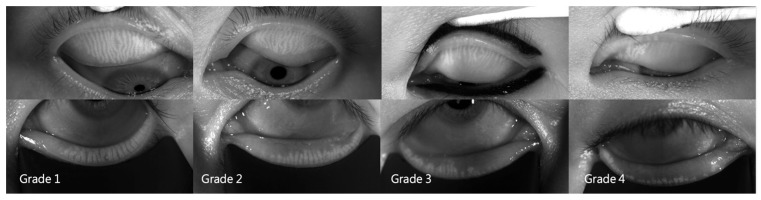
Representative meibography images of the four meiboscale grades.

A LipiView^®^ II interferometer (Johnson & Johnson Vision, Jacksonville, FL, USA) was used to measure LLT and total/incomplete blinks per 20 s and to perform meibography. The number of total blinks was the sum of incomplete and complete blinks. The incomplete blink rate was the number of incomplete blinks ÷ number of total blinks × 100%. The number of expressible meibomian glands (MGEs) was determined using a handheld Meibomian Gland Evaluator^™^ (TearScience, Morrisville, NC, USA). The instrument was applied to the nasal, central, and temporal regions of the upper and lower eyelids, and 5–6 meibomian glands were determined in each region (i.e., 15–18 glands in both the upper and lower lids). The number of MGEs was counted using slit-lamp biomicroscopy. The total number of MGEs from the upper and lower eyelids was used for further analysis. The upper- and lower-eyelid MGEs were used for further analysis. After capturing the meibomian gland images using the LipiView^®^ II, the extent of meibomian gland dropout was graded according to the meiboscale [27]. The meiboscale grades of each eyelid were 0–4 (grade 0, no gland loss; grade 1, ≤25% area of gland loss; grade 2, 26–50% area of gland loss; grade 3, 51–75% area of gland loss; grade 4, >75% area of gland loss) by one well-trained examiner based on meibography (Figure 2). The meiboscale grades of the upper and lower eyelids were determined separately. The Schirmer test II with anesthetics and a standard 35 × 5-mm tear test strip (Eagle Vision, Katena Products, Memphis, TN, USA) were used to measure aqueous tear secretion at 5 min.

### 2.3. Measurement of Fluorescein Tear-Film Break-Up Time (FTBUT) and Grading of Superficial Punctate Keratitis (SPK)

**Figure 3:**
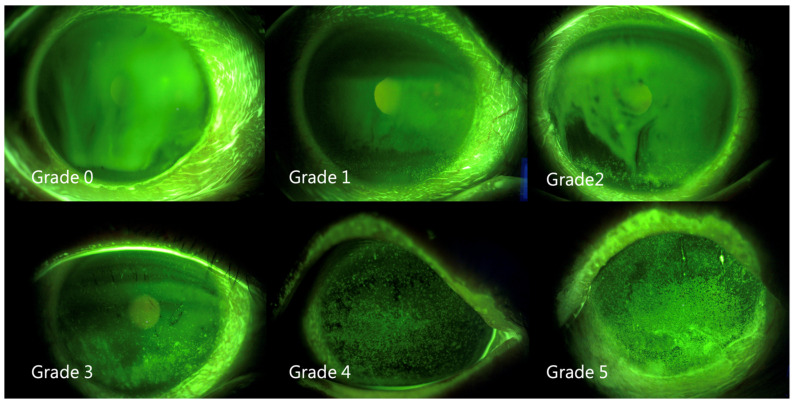
Representative external eye photographs of fluorescein stain.

The FTBUT was measured (average of three) after applying fluorescein solution on the bulbar conjunctiva, as previously described [28]. The corneal/conjunctival staining patterns were graded by an ophthalmologist from 0 to 4 according to the Oxford scheme (Figure 3) [29].

### 2.4. Statistical Analysis

All statistical analyses were performed using SPSS v.20.0 (IBM SPSS Statistics for Windows, IBM Corp., Armonk, NY, USA). All numeric variables were assessed for normality using the Kolmogorov–Smirnov test. The Kruskal–Wallis test was used to compare the numeric variables, including the SPEED questionnaire score, OSDI questionnaire score, LLT, Schirmer test results, meiboscale grades, number of MGEs, and total/incomplete blinks, among the DM groups. Descriptive results are presented as median [Q1–Q3]. Since most parameters were not normally distributed, Spearman’s rank correlation coefficient was used to determine the correlations between relevant parameters. To remove potential confounding effect, age-adjustment to DM severity was also conducted. The chi-square test was used to examine the sex distribution. Differences in parameters before and after lid hygiene were evaluated using the Friedman test. Differences in parameters of the 1st and 2nd examination in patients that did not perform lid hygiene were also evaluated using the Friedman test. *p* values of <0.05 were considered statistically significant.

## 3. Results

### 3.1. Demographics

The median age of the 2328 patients was 55.3 [46.0–66.0] years. Patients with more severe dermatochalasis were significantly older (*p* < 0.001) (Table 1A). There were 1770 females and 558 males, with a female predominance (76.0%) (Table 1B). Females were significantly older (58.0 [47.0–66.0] years) than males (55.0 [41.5–65.0] years) (*p* < 0.001). However, the females had less severe dermatochalasis (Table 1B, chi-square *p* = 0.012), and the odds ratio of males with dermatochalasis >DM-3 was 1.375 (*p* = 0.004).

### 3.2. Dermatochalasis Severity and Subjective Symptoms

The 2328 patients had a median SPEED questionnaire score of 12.0 [8.0–16.0] and a median OSDI questionnaire score of 37.5 [25.0–54.9]. Patients with more severe dermatochalasis were less symptomatic (Table 2A). The differences among severity groups were more significant in the SPEED questionnaire than OSDI results. Dermatochalasis severity correlated positively with patient age and negatively with symptom parameters (Table 2B). The correlation between dermatochalasis severity and symptoms was more noticeable with SPEED than with OSDI. The correlation was greater between the Schirmer test and OSDI (*r_s_* = −0.070, *p* = 0.001) than between the Schirmer test and SPEED (*r*_s_ = −0.044, *p* = 0.034).

### 3.3. Dermatochalasis Severity and Meibomian Parameters

Patients with more severe dermatochalasis had less MGE and high meiboscale grades, i.e., less well-functioning meibomian gland (MG) (*p* = 0.022) and more MG structural loss (Table 3A) (*p* < 0.001). The median meiboscale grade was greater for upper lids. Paradoxically, patients with more severe dermatochalasis also had higher average LLT (*p* < 0.001). The severity of dermatochalasis correlated positively with LLT and meiboscale grade but negatively with MGE (Table 3B). The correlations between dermatochalasis severity and meiboscale grade as well as between dermatochalasis severity and MGE were more significant for the upper lid. LLT correlated positively with MGE for both the upper and lower lids, although more significantly for the lower lids. LLT correlated negatively with meiboscale grade, but only with that of the lower lid (Table 3B). MGE correlated with meiboscale, slightly more for the upper lid (*r*_s_ *=* −0.210 and *p* < 0.001 for the upper lids vs. *r*_s_ *=* −0.186 and *p* < 0.001 for the lower lids). The proportion of patients with LLT ≥ 100 nm was 18.3% (Table 4). The proportion of LLT ≥ 100 increased significantly from DM-1 to DM-4 (chi-square *p* < 0.001). The odds ratio of having LLT ≥ 100 was 2.06 (*p* < 0.001) and 2.59 (*p* < 0.001) for patients with dermatochalasis more severe than DM-3 and DM-4, respectively.

### 3.4. Dermatochalasis Severity and Tear-Film/Blink Parameters

Patients with greater dermatochalasis severity had fewer partial blinks and total blinks (Table 5A). However, there was no significant difference in the Schirmer test, FTBUT, and SPK results among the dermatochalasis severity groups. Patient age correlated negatively with the number of total blinks, partial blinks, partial blink rate, Schirmer test results, and FTBUT results (Table 5B). There was also a positive correlation between the number of total blinks and SPEED/OSDI questionnaire scores (*r*_s_ = 0.111, *p* < 0.001 and *r*_s_ = 0.070, *p* = 0.001, respectively). The number of total blinks correlated negatively with LLT (*r*_s_ = −0.124, *p* < 0.001). The number of partial blinks and the partial blink rate negatively correlated with meiboscale grade (r_s_ = −0.051, *p* = 0.015 and r_s_ = −0.052, *p* = 0.014, respectively).

### 3.5. Differences in High LLT Distribution between Females and Males

There was a significantly greater proportion of females with an LLT ≥ 100 (20.3% vs. 11.6%, *p* < 0.001) (Table 6). The odds ratio of females having an LLT ≥ 100 was 1.746.

### 3.6. Effects of Lid Hygiene

The compliance rate of follow-up examination at 3 months was 27.7% (644 out of 2328 patients). For the 644 patients who performed lid hygiene and underwent follow-up examinations, lid hygiene significantly improved subjective symptoms (Table 7A) and decreased LLT (Table 7B). However, lid hygiene did not change the number of MGEs, meiboscale grade, Schirmer test results, or FTBUT (*p* = 0.647, 0.180, 0.262, and 0.405, respectively) (Table 7B). For the 238 patients who did not perform lid hygiene but had undergone follow-up examinations, improvement in subjective symptoms was noticed only in OSDI (B) (Table 8A). There was little improvement in objective parameters (Table 8B) between the 1st and 2nd examination.

## 4. Discussion

In the evaluation of tear-film lipid-related parameters, we regarded LLT as a short-term indicator, MGE as an intermediate indicator, and the severity of structural loss (meiboscale grade) as a long-term indicator. Meibomian dropout, as a diagnostic parameter for MGD, has a sensitivity of 96.7% and a specificity of 85% [20]. Patients with more severe dermatochalasis also had higher meiboscale grades (Table 3), which was higher for the upper lids (Table 6A) whose MG orifices are closer to the dermatochalasis overhanging skin. Thus, we suggest that dermatochalasis could contribute to MG loss severity. Redundant skin could have hindered eyelid cleaning, and accumulated sebum could have induced inflammation of the MG orifice, especially in the upper eyelids, which resulted in functional obstruction of the MG with less MGE at the intermediate stage and structural loss with higher meiboscale grades at the late stage.

Our results are similar to those of previous studies showing that the LLT increases with age [11,18] and is significantly correlated with both meibomian gland expressibility and morphology in obstructive MGD [14,18,19]. The positive association between MGE and LLT (Table 3B) indicated that fewer secreting meibomian glands would lead to a lower LLT. Increased meibomian gland structural loss would also result in less secreted meibum. Theoretically, there should be a negative association between meiboscale grade and LLT. However, LLT correlated negatively with meiboscale grade only in the lower lid in our study. More than one third of DM-4 patients had an LLT ≥ 100 nm (Table 4), and the odds ratio of having an LLT ≥ 100 was 2.59 (*p* < 0.001) for patients with DM-4. As DM-4 patients had a median meiboscale grade of 1.5, sources of lipid other than meibum should have contributed to the measured LLT. Since sebum also contributes to the tear-film lipid-layer [30], other lipids may have been measured by the interferometry. Sebum in the dermatochalasis skin folds may have contributed to the increased LLT. This paradoxical LLT measurement result confounded the negative correlation between meiboscale grade and LLT of the upper lid. Eyelid cleaning 1 day before interferometry could potentially reduce the confounding effect.

We found a significant positive correlation between meiboscale grade and age (Table 3B), which is consistent with a report that MG dropout increased with age [11]. Our results also showed a negative association between MGE and age (Table 3B). Both results implied fewer secreting glands, and thus, lower LLT in older patients. However, the LLT correlated positively with age in our study (Table 3B), but these seemingly contradictory results have been seen previously [11,18,21]. This confounding effect could also have been caused by dermatochalasis-related effects.

Given that dermatochalasis increases with age [3] and females were older in our included patients, they should have had more severe dermatochalasis. However, males had more severe dermatochalasis, and the odds ratio of males, compared with females, having dermatochalasis > DM-3 was 1.375 (*p* = 0.004), probably because double-eyelid surgery is more commonly performed among females in Taiwan.

Since the dermatochalasis severity was less in females (Table 1B), there should have been less dermatochalasis-related thick LLT and thinner LLT. For all of the 2328 included patients, LLT was higher in females, consistent with a previous study finding that females had thicker LLT [21]. More females also had an LLT ≥ 100 nm (Table 6B). These seemingly contradictory results could have been caused by the periocular cosmetics commonly used by females. These cosmetics contain oil components but were incompletely cleaned. When lid hygiene was performed, both LLT and subjective symptoms decreased, although there were no changes in the intermediate indicator MGE and long-term indicator meiboscale grade (Table 7). The improvement was less significant in the more severe DM-3 and DM-4 patients (Table 7), which further confirmed that dermatochalasis interferes with regular lid hygiene, perpetuating the smoldering inflammation of the MG orifices, and causing intermediate functional obstruction of MGE and long-term progressive loss of MG.

For a cutoff value of ≤75 nm LLT, the sensitivity was 65.8% and the specificity was 63.4% for detecting MGD. With a cutoff value of ≤60 nm, the sensitivity was 47.9% and the specificity was 90.2% [31]. In our study, 58.8% of all included patients had an LLT of ≤75 nm, whereas 51.5% of the DM-4 patients had an LLT of ≤75 nm. Furthermore, 37.8% of all included patients had an LLT of ≤60 nm, whereas 31.8% of the DM-4 patients had an LLT of ≤60 nm. Using these cutoff values could have missed a great proportion of patients with MGD in our scenario. The severity of dermatochalasis should be considered when interpreting LLT results. Performing a second examination after better lid hygiene would enhance the accuracy of meibomian gland-related assessments.

Dermatochalasis severity correlated negatively with symptoms (Table 2B). This finding could result from the lower corneal sensitivity in older patients [32] because the patients with more severe dermatochalasis were older and showed negative correlations among age and the SPEED and OSDI scores.

Dermatochalasis severity correlated positively with LLT (Table 2B). LLT could be considered as the amount of secreted lipid divided by the area of distribution. It is possible that the smaller lid fissure, and thus the smaller surface area to be covered by the secreted meibum, resulted in a thicker LLT in patients with severe dermatochalasis. If true, lid hygiene should not change the LLT since it did not change the MGE and meiboscale grade, indicating that the amount of meibum secretion was not changed. In contrast, about 10% of the tear-film lipids are from sebum. It is more reasonable that an excessive amount of sebum in the dermatochalasis skin folds contributed more lipids to the tear-film and manifested as a thicker LLT. Removal of excessive periocular sebum by performing lid hygiene would thus reduce LLT. In this study, we confirmed that lid hygiene significantly decreased LLT, but not MGE and meiboscale grade. This confirmed that thick LLT in patients with severe dermatochalasis should have resulted at least partially from an abnormally large amount of sebum.

The severity of dermatochalasis correlated positively with meiboscale grade and negatively with MGE (Table 3B), with stronger upper lid correlation. This is reasonable since the dermatochalasis is close to the upper lids. Lid hygiene-related inflammation and subsequent functional obstruction of the meibomian orifice would thus be more prominent in the upper lids. In contrast, LLT correlated positively with MGE in both the upper and lower lids but negatively with meiboscale grade only in the lower lid (Table 3B). These findings are also conceivable as the LipiView II^®^ interferometer measures LLT in the lower tear meniscus. The correlation coefficients were greater between dermatochalasis and meiboscale grades than between LLT and meiboscale grades, which also indicated that the long-term impact of dermatochalasis on eyelid structures was more significant than the short-term LLT fluctuation.

Blinking is associated with orbicularis oculi and meibum secretion [7,8]. The orbicularis oculi muscle fibers become thin [5] with loss of muscle strength and function in the older population [6]. These changes decrease orbicularis oculi muscle function, which decreases the blink efficacy and leads to meibum stagnation, thus further causing MGD and meibomian gland atrophy [8].

Meibomian gland atrophy results in tear-film instability and subsequently leads to more dry-eye symptoms and a high blinking rate [11,33,34], which is a compensatory mechanism commonly observed in patients with dry-eye disease and is triggered by reduced tear-film stability and the resulting discomfort [33,34]. Our results showing a positive correlation between the number of total blinks and SPEED/OSDI questionnaire scores confirmed that the number of total blinks is associated with subjective discomfort [11,35]. This could be a compensatory mechanism to protect against progressive meibomian gland loss.

There were fewer partial blinks in patients with more severe dermatochalasis (Table 5). The smaller lid fissures in these patients possibly made complete blinking easier because the excursion of the orbicularis muscles for a complete blink was shorter. Similarly, there was less partial blinking in males, which also could have resulted from the more severe dermatochalasis (i.e., smaller lid fissures) in males.

Only 27.7% (644/2328) of all enrolled patients were lid hygiene compliant for 3 months and completed comprehensive examinations. Among them, lid hygiene significantly decreased LLT (Table 7B), indicating that the LLT contained meibum from the MG and from skin sebum, which could be removed by lid hygiene. However, the improvement was less significant in patients with more severe dermatochalasis, emphasizing the importance of lid folds on lid hygiene. Lid hygiene also did not change the MGE and meiboscale grade (*p* = 0.127 and 0.740, respectively), suggesting that subjective symptoms and LLT were short-term indicators of dry eyes, and both improved quickly with lid hygiene. In contrast, functional obstruction of MG took longer to improve [23,24] and was an intermediate indicator of MGD. The structural changes in meiboscale grade were even more permanent and were long-term indicators of MGD. In a study, meibomian gland atrophy was unchanged 6 months after thermal pulsation treatment [22]; however, another study reported an improvement [23]. In contrast, the decrease in Schirmer test results (*p* < 0.001) and increase in FTBUTs (*p* = 0.004) after lid hygiene in our study indicated decreased reflex tearing and stabilization of tear-film when the abnormally excessive sebum and/or meibum were cleared by lid hygiene.

The study strength was the large sample of patients who visited a single ophthalmologist at the dry-eye center. Moreover, the treatment rationale and patient-education program remained consistent throughout the study period. We identified dermatochalasis as a factor contributing to the severity of MGD. However, the correlation was weak, with a low correlation coefficient. This is similar to a previous study that reported non-significant or weakly significant correlations between clinical parameters [18], with correlation coefficients of <0.2. This could be attributed to the multifactorial nature of dry-eye disease [36] and the discordance between subjective symptoms and objective signs [14]. In addition, structural changes in the meibomian glands are a result of chronic MGD rather than a short-term effect, such as after cataract/corneal surgery [37,38,39] and lid hygiene, as shown in this study. The temporal effects on the change in LLT, MGE, and meiboscale grade vary, which may contribute to the disparity that is frequently encountered in dry-eye studies.

One limitation is the retrospective nature of our study. Patient dropout due to suboptimal compliance is a major issue. However, the results reflected a real-world scenario and are thus readily applicable to most daily practices. Another limitation is that we defined compliance as returning to the clinic for follow-up examinations, but lid hygiene possibly was not fully performed. Thus, the true lid hygiene compliance rate could have been overestimated. In contrast, some patients with good compliance may have improved so much that they did not return for further follow-up examinations, which would have led to an underestimation of compliance. Another limitation is that we did not include systemic conditions in the analysis. Patients with systemic diseases showed more severe dry-eye disease signs [15], whereas patients with lower self-perceived health had higher discordance between symptoms and signs [13]. Not including these factors in this study could have led to the low correlation coefficients. A third limitation is that we did not recruit patients with less severe dry eye symptoms as they were encouraged not to visit tertiary medical center in our health care system. Further study to enroll patients with less severity in the general ophthalmology clinics to verify the association between DM severity and MGD would facilitate the generalization.

## 5. Conclusions

Dermatochalasis contributes to a substantial proportion of dry-eye pathophysiology, particularly MGD. If not detected and managed early, chronic inflammation might lead to meibomian gland orifice obstruction and subsequent atrophy.

## Figures and Tables

**Table 1:** Summary of patients’ demographic characteristics. (A) Summary of patient age. (B) Distribution of dermatochalasis in females and males.

(A)								
		Age (Years)						
	N	Median	Q1	Q3	Minimum	Maximum		*p*
DM-1	917	50.9	41.0	62.0	20.0	90.0		**<0.001**
DM-2	880	53.9	44.0	64.8	20.0	90.0		
DM-3	304	63.5	57.0	70.0	29.0	93.0		
DM-4	227	66.8	61.0	74.0	36.0	91.0		
Total	2328	55.3	46.0	66.0	20.0	93.0		
**(B)**								
**Sex**	**Female**		**Male**			**Total**		
**Group**	**N**	**% within Female**	**N**	**% within Male**		**N**	**%**	***p***
DM-1	712	40.3%	205	36.7%		917	39.4%	**0.012**
DM-2	679	38.3%	201	36.0%		880	37.8%	
DM-3	209	11.8%	95	17.0%		304	13.1%	
DM-4	170	9.6%	57	10.2%		227	9.7%	
Total	1770	100.0%	558	100.0%		2328	100.0%	

N: number of patients in the indicated group; Q1: lower quartile; Q3: upper quartile; *p*: statistical significance using the Kruskal–Wallis test (A) or Pearson’s chi-square test (B).

**Table 2:** Summary and correlation of subjective symptoms (A).Summary of subjective symptoms according to dermatochalasis severity. (B) Correlations among dermatochalasis severity, age, and subjective symptom parameters.

(A)						
	DM-1	DM-2	DM-3	DM-4	Total	*p*
N	917	880	304	227	2328	
SPEED	12.0 [8.0–16.0]	12.0 [8.0–16.0]	11.0 [7.0–15.0]	10.0 [6.0–15.0]	12.0 [8.0–16.0]	**0.001**
Frequency (SPEED)	5.0 [4.0–7.0]	5.0 [4.0–7.0]	5.0 [3.0–7.0]	5.0 [3.0–7.0]	5.0 [4.0–7.0]	**0.001**
Severity (SPEED)	6.0 [4.0–9.0]	6.0 [4.0–8.0]	6.0 [3.0–8.0]	5.0 [3.0–8.0]	6.0 [4.0–8.0]	**0.003**
OSDI	37.5 [25.0–54.9]	37.0 [22.5–56.3]	33.3 [20.6–54.8]	36.8 [18.8–55.4]	36.4 [22.7–55.6]	**0.036**
OSDI (A)	8.0 [5.0–11.0]	8.0 [5.0–12.0]	8.0 [4.0–12.0]	8.0 [4.0–12.0]	8.0 [5.0–12.0]	0.984
OSDI (B)	4.0 [2.0–8.0]	4.0 [2.0–8.0]	4.0 [2.0–7.0]	4.0 [1.0–7.0]	4.0 [2.0–8.0]	**0.007**
OSDI (C)	3.0 [2.0–7.0]	3.0 [1.0–6.0]	3.0 [1.0–6.0]	3.0 [0.0–5.0]	3.0 [1.0–7.0]	**<0.001**
**(B)**						
	**DM**		**Age**		**Age Adjusted DM**	
	***r_s_***	***ρ***	***r_s_***	***ρ***	***r_s_***	***ρ***
Age	0.351	**<0.001**	1			
SPEED	−0.085	**<0.001**	−0.149	**<0.001**	0.053	**0.005**
Frequency (SPEED)	−0.076	**0.001**	−0.133	**<0.001**	0.064	**0.001**
Severity (SPEED)	−0.085	**<0.001**	−0.147	**<0.001**	0.041	**0.029**
OSDI	−0.041	0.076	0.016	0.487	0.040	**0.036**
OSDI (A)	−0.026	0.273	0.077	**0.001**	0.054	**0.004**
OSDI (B)	−0.063	**0.007**	−0.111	**<0.001**	0.033	0.077
OSDI (C)	−0.110	**<0.001**	−0.153	**<0.001**	−0.005	0.810

DM: dermatochalasis (grade); numbers in brackets represent the 1st and 3rd quartiles; *r_s_*: correlation coefficient by Spearman’s test. *p:* statistical significance using the Kruskal–Wallis test (A) or Spearman’s test (B).

**Table 3:** Summary and correlations of meibomian gland-associated parameters. (A)Summary of meibomian gland-associated parameters according to dermatochalasis severity. (B) Correlations between dermatochalasis severity and lipid-related parameters.

(A)									
	DM-1	DM-2	DM-3		DM-4			Total	*p*
N	917	880	304		227			2328	
LLT (nm)	66.5 [56.0–86.0]	69.0 [58.0–88.0]	76.0 [58.0–97.0]		81.0 [63.0–110.0]			69.0 [57.0–91.0]	**<0.001**
MGE	8.0 [5.0–11.0]	8.0 [5.0–11.0]	8.0 [5.0–10.0]		7.0 [5.0–10.0]			8.0 [5.0–10.0]	**0.022**
MGE (upper)	5.0 [3.0–6.0]	5.0 [3.0–6.0]	4.0 [2.0–6.0]		4.0 [2.0–6.0]			5.0 [3.0–6.0]	**<0.001**
MGE (lower)	3.0 [2.0–5.0]	3.0 [2.0–5.0]	3.0 [2.0–5.0]		3.0 [2.0–5.0]			3.0 [2.0–5.0]	0.657
Meiboscale (grade)	1.0 [1.0–1.5]	1.0 [1.0–1.5]	1.5 [1.0–2.0]		1.5 [1.0–2.0]			1.5 [1.0–2.0]	**<0.001**
Meiboscale (upper)	1.0 [1.0–2.0]	1.0 [1.0–2.0]	2.0 [1.0–2.0]		2.0 [1.0–3.0]			1.0 [1.0–2.0]	**<0.001**
Meiboscale (lower)	1.0 [1.0–1.0]	1.0 [1.0–1.0]	1.0 [1.0–2.0]		1.0 [1.0–2.0]			1.0 [1.0–1.0]	**0.003**
**(B)**									
		**DM (Grade)**	**Age (Years)**			**Age Adjusted DM**		**LLT (nm)**	
		***r_s_***	***ρ***	***r_s_***	***r_s_***	***r_s_***	***ρ***	***r_s_***	***ρ***
LLT		0.122	**<0.001**	0.178	**<0.001**	0.043	**0.022**	1	
MGE	Total	−0.040	0.051	−0.074	**<0.001**	−0.005	0.781	0.117	**<0.001**
	upper	−0.064	**0.002**	−0.052 ^*^	**0.012**	−0.014	0.462	0.075	**<0.001**
	lower	0.000	0.991	−0.066	**0.001**	0.000	0.988	0.117	**<0.001**
Meiboscale	Total	0.096	**<0.001**	0.278	**<0.001**	0.040	**0.034**	−0.01	0.626
	upper	0.100	**<0.001**	0.297	**<0.001**	0.057	**0.003**	0.003	0.896
	lower	0.033	0.111	0.122	**<0.001**	0.011	0.560	−0.052	**0.012**

DM: dermatochalasis; LLT: average lipid-layer thickness; MGE: number of expressible meibomian glands; numbers in brackets represent 1st and 3rd quartiles; *r_s_*: Spearman’s correlation coefficient; *p:* statistical significance using the Kruskal–Wallis test (A) or Spearman’s test (B).

**Table 4:** Distribution of LLT ≥ 100 nm among the dermatochalasis groups.

		LLT < 100	LLT ≥ 100	Total	*p*
DM-1	N	774	144	918	<0.001
	% within DM-1	84.3%	15.7%	100.0%	
DM-2	N	744	135	879	
	% within DM-2	84.6%	15.4%	100.0%	
DM-3	N	235	69	304	
	% within DM-3	77.3%	22.7%	100.0%	
DM-4	N	150	77	227	
	% within DM-4	66.1%	33.9%	100.0%	
Total	N	1903	425	2328	
	% within DM	81.7%	18.3%	100.0%	

LLT: average lipid-layer thickness (nm); DM: dermatochalasis; N: number of patients in the indicated group; *p*: statistical significance using Pearson’s chi-square test.

**Table 5:** Summary and correlation of tear-film, blink patterns, and corneal parameters (A). Summary of tear-film, blink patterns, and corneal parameters according to dermatochalasis severity (B) Correlations among dermatochalasis, age, blinks, and tear-film parameters.

(A)										
	DM-1	DM-2	DM-3		DM-4		Total		*p*	
N	917	880	304		227		2328			
PB	3.0 [1.0–6.0]	3.0 [1.0–7.0]	3.0 [1.0–5.0]		2.0 [1.0–5.0]		3.0 [1.0–6.0]		**<0.001**	
PB (%)	62.5 [27.3–92.9]	66.7 [33.3–90.0]	63.6 [26.8–91.3]		50.0 [20.0–84.2]		62.5 [28.6–90.9]		0.106	
TB	7.0 [4.0–11.0]	7.0 [4.0–10.0]	6.0 [3.0–8.0]		6.0 [3.0–9.0]		6.0 [4.0–10.0]		**<0.001**	
Schirmer (mm)	4.0 [2.0–7.0]	4.0 [2.0–6.0]	4.0 [2.0–7.0]		4.0 [2.0–7.0]		4.0 [2.0–7.0]		0.776	
TBUT (s)	3.0 [2.0–3.0]	3.0 [2.0–3.0]	3.0 [2.0–3.0]		2.5 [2.0–3.0]		3.0 [2.0–3.0]		0.284	
SPK (grade)	0.0 [0.0–0.0]	0.0 [0.0–0.0]	0.0 [0.0–0.0]		0.0 [0.0–0.0]		0.0 [0.0–0.0]		0.417	
**(B)**										
	**DM (Grade)**		**Age (Year)**		**A** **ge Adjusted DM**		**Schirmer (mm)**		**SPK (Grade)**	
	***r_s_***	***ρ***	***r_s_***	***ρ***	***r_s_***	***ρ***	***r_s_***	***ρ***	***r_s_***	***ρ***
TB	−0.115	**<0.001**	−0.218	**<0.001**	−0.087	**<0.001**	−0.019	0.416	0.017	0.471
PB	−0.085	**<0.001**	−0.194	**<0.001**	−0.054	**0.004**	−0.044	0.059	0.040	0.091
PB (%)	−0.011	0.628	−0.076	**0.001**	−0.015	0.438	−0.030	0.213	0.026	0.270
Schirmer (mm)	0.008	0.743	−0.065	**0.005**	−0.017	0.362	1		−0.073	**0.002**
FTBUT (s)	−0.027	0.251	−0.094	**<0.001**	−0.020	0.290	0.151	**<0.001**	−0.151	**<0.001**
SPK (grade)	−0.005	0.828	0.038	0.100	0.022	0.257	−0.073	0.002	1	

DM: dermatochalasis; MGE: number of expressible meibomian glands; PB: number of partial blinks; TB: total number of blinks; PB (%): partial blink rate; FTBUT: fluorescein tear-film break-up time; SPK: superficial punctate keratitis; numbers in brackets represent 1st and 3rd quartiles; *r_s_*: Spearman’s correlation coefficient; *p*: statistical significance using the Kruskal–Wallis test (A) or Spearman’s test (B).

**Table 6:** Distribution of high LLT in females and males.

N (% within Group)	Female	Male	Total	*p*
LLT < 100	1410 (79.70%)	493 (88.40%)	1903 (81.70%)	<0.001
LLT ≥ 100	360 (20.30%)	65 (11.60%)	425 (18.30%)	
Total	1770	558	2328	

N: case number of the indicated group; *p:* statistical significance by Chi–square test.

**Table 7:** Summary of subjective and objective dry-eye parameters before and after lid hygiene. (A) Summary of subjective symptoms before and after lid hygiene. (B) Summary of objective signs before and after lid hygiene.

(A)																
		DM 1–4 (N = 644)			DM-1 (N = 223)			DM-2 (N = 279)			DM-3 (N = 95)			DM4 (N = 47)		
		Median	IQR	*p*	Median	IQR	*p*	Median	IQR	*p*	Median	IQR	*p*	Median	IQR	*p*
SPEED (total)	Before	12.0	8.0	**<0.001**	12.0	8.0	**<0.001**	12.0	8.0	**<0.001**	11.80	7.0	**<0.001**	12.0	7.00	**0.007**
	After	8.0	7.0		9.0	8.0		8.0	6.0		9.0	7.0		9.0	8.00	
Frequency (SPEED)	Before	5.0	3.0	**<0.001**	6.0	4.0	**<0.001**	6.0	3.0	**<0.001**	5.57	3.0	**0.002**	6.0	4.00	**0.031**
	After	4.0	3.0		4.0	4.0		4.0	2.0		4.0	3.0		4.0	3.00	
Severity (SPEED)	Before	6.0	4.0	**<0.001**	6.0	5.0	**<0.001**	6.0	4.0	**<0.001**	6.23	5.0	**<0.001**	6.0	5.00	**0.008**
	After	4.0	5.0		5.0	5.0		4.0	3.0		5.0	4.0		5.0	5.00	
OSDI (total)	Before	36.4	33.5	**<0.001**	35.4	33.3	**<0.001**	37.5	31.8	**<0.001**	41.70	35.8	**0.002**	45.5	46.53	**0.018**
	After	25.0	26.7		25.0	25.0		27.1	25.0		29.4	33.5		31.3	26.45	
OSDI (A)	Before	7.0	6.0	**<0.001**	8.0	6.0	**<0.001**	8.0	6.0	**<0.001**	8.54	8.0	**0.122**	10.0	9.00	**<0.001**
	After	5.0	5.0		5.0	5.0		5.0	5.0		6.0	8.0		6.0	6.00	
OSDI (B)	Before	4.0	5.8	**<0.001**	4.0	4.0	**<0.001**	4.0	5.0	**<0.001**	5.38	6.0	**0.004**	4.0	7.00	0.139
	After	3.0	4.0		3.0	4.0		3.0	4.0		3.0	5.0		4.0	6.00	
OSDI (C)	Before	3.0	4.8	**<0.001**	3.0	5.0	**<0.001**	3.0	5.0	**0.006**	4.06	5.0	**0.001**	3.0	6.00	0.194
	After	3.0	4.0		3.0	5.0		3.0	4.0		3.0	5.0		3.0	5.00	
**(B)**																
		**DM 1–4 (N = 644)**			**DM-1 (N = 223)**			**DM-2 (N = 279)**			**DM-3 (N = 95)**			**DM-4 (N = 47)**		
		**Median**	**IQR**	***p***	**Median**	**IQR**	***p***	**Median**	**IQR**	***p***	**Median**	**IQR**	***p***	**Median**	**IQR**	***p***
LLT (nm)	Before	63.0	35.0	**0.004**	64.0	32.0	**0.003**	67.0	37.0	**0.004**	69.0	38.0	0.159	73.0	53.0	0.105
	After	63.0	29.0		62.0	27.3		65.0	28.0		68.0	34.0		61.5	32.0	
MGE	Before	7.0	5.0	0.127	7.0	6.0	0.260	8.0	4.0	0.901	8.0	5.0	0.453	8.0	6.0	0.647
	After	8.0	5.0		8.0	6.0		8.0	5.0		8.0	4.0		8.0	5.0	
Meiboscale(grade)	Before	1.0	1.0	0.740	1.5	1.0	1.000	1.5	0.5	0.414	1.5	1.0	0.782	2.0	1.0	0.180
	After	1.0	1.0		1.5	1.0		1.5	0.5		1.5	1.0		1.5	1.0	
PB	Before	3.0	5.0	**<0.001**	3.0	5.0	**<0.001**	4.0	6.0	**<0.001**	3.0	5.0	**0.022**	2.0	5.0	0.355
	After	2.0	4.0		2.0	4.0		2.0	4.0		2.0	4.0		2.0	5.3	
PB (%)	Before	64.3	67.9	**<0.001**	50.0	65.9	**0.008**	66.7	59.0	**<0.001**	71.4	70.8	**0.002**	50.0	76.8	0.128
	After	40.0	65.0		40.0	65.0		40.0	68.6		40.0	75.8		33.3	67.1	
TB	Before	7.0	7.0	0.627	7.0	8.0	0.532	7.0	7.0	0.754	6.0	6.0	1.000	6.0	4.0	0.435
	After	7.0	7.0		7.0	7.0		7.0	6.0		7.0	8.0		7.0	6.3	
Schirmer test (mm)	Before	4.0	5.0	**<0.001**	4.0	6.0	**0.028**	4.0	5.0	**0.002**	4.0	5.0	0.138	4.0	5.0	0.262
	After	4.0	4.0		3.0	5.0		3.0	4.3		4.0	4.0		4.0	6.0	
FTBUT (sec)	Before	3.0	1.0	**0.007**	3.0	1.0	0.152	3.0	1.0	0.204	3.0	1.0	0.072	2.0	1.0	0.405
	After	3.0	1.0		3.0	2.0		3.0	2.0		3.0	1.0		3.0	1.0	
SPK (grade)	Before	0.0	0.0	0.103	0.0	0.0	0.206	0.0	0.0	0.895	0.0	0.0	0.197	0.0	0.0	0.102
	After	0.0	0.0		0.0	0.0		0.0	0.0		0.0	0.0		0.0	0.0	

DM: dermatochalasis; IQR: interquatile range; MGE: number of expressible meibomian glands; OSDI (A): subtotal of the frequency of symptoms; OSDI (B): subtotal of the frequency of activity limitation; OSDI (C): subtotal of the frequency of environmental factors triggering discomfort; LLT: average lipid-layer thickness; PB: number of partial blinks; B (%): partial blink rate; TB: total number of blinks; FTBUT: fluorescein tear-film break-up time; SPK: superficial punctate keratitis; *p*: statistical significance using the Friedman test.

**Table 8:** Summary of subjective and objective dry-eye parameters in patients who did not perform lid hygiene. (A) Summary of subjective symptoms of 1st and 2nd examination. (B) Summary of objective signs of 1st and 2nd examination.

(A)																
		DM 1–4 (N = 238)			DM-1 (N =103)			DM-2 (N = 83)			DM-3 (N = 31)			DM-4 (N = 21)		
	Exam	Median	IQR	*p*	Median	IQR	*p*	Median	IQR	*p*	Median	IQR	*p*	Median	IQR	*p*
SPEED (total)	1st	11.0	8.0	0.087	12.0	8.0	0.179	11.0	7.0	0.265	10.0	7.0	0.883	10.0	8.0	0.458
	2nd	10.0	8.0		10.0	8.0		11.0	8.0		10.0	9.0		7.0	7.0	
Frequency (SPEED)	1st	5.0	3.0	0.062	5.0	3.0	0.312	5.0	3.0	0.154	4.0	3.0	0.726	5.0	3.0	0.308
	2nd	4.5	4.0		5.0	4.0		5.0	2.0		4.0	4.0		4.0	3.5	
Severity (SPEED)	1st	6.0	4.0	0.149	6.0	4.5	0.155	6.0	4.0	0.437	5.0	5.0	0.980	5.0	5.0	0.726
	2nd	5.0	5.0		5.0	5.0		5.0	5.0		5.0	7.0		4.0	4.5	
OSDI(total)	1st	36.4	33.1	0.091	37.5	30.0	0.305	36.0	34.3	0.305	31.8	32.7	0.595	32.5	34.0	0.498
	2nd	31.3	31.5		31.3	38.9		31.8	27.5		32.5	22.2		29.2	24.9	
OSDI (A)	1st	8.0	6.3	0.093	8.0	6.5	0.180	8.0	8.0	0.506	7.0	7.0	0.498	7.0	8.0	0.539
	2nd	7.0	7.0		7.0	7.0		7.0	7.0		6.0	5.0		5.0	8.0	
OSDI (B)	1st	4.0	5.0	**<0.001**	4.0	6.0	**0.049**	4.0	5.0	**0.024**	3.0	4.0	0.070	3.0	5.0	0.378
	2nd	3.0	5.0		4.0	5.0		3.0	3.0		3.0	4.0		2.0	3.5	
OSDI (C)	1st	3.0	5.0	0.727	3.0	5.5	0.881	3.0	5.0	0.841	3.0	5.0	0.381	3.0	5.0	0.324
	2nd	3.0	4.3		3.0	4.0		3.0	5.0		3.0	7.0		3.0	2.5	
**(B)**																
		**DM 1–4 (N = 238)**			**DM-1 (N =103)**			**DM-2 (N = 83)**			**DM-3 (N = 31)**			**DM-4 (N = 21)**		
	**Exam**	**Median**	**IQR**	***p***	**Median**	**IQR**	***p***	**Median**	**IQR**	***p***	**Median**	**IQR**	***p***	**Median**	**IQR**	***p***
LLT (nm)	1st	69.0	33.0	0.368	66.0	28.0	0.797	69.0	29.0	0.244	77.0	38.0	0.748	86.0	48.0	0.370
	2nd	68.5	36.0		64.0	35.0		67.0	29.0		74.0	34.0		83.0	42.5	
MGE	1st	8.0	6.0	0.720	8.0	5.0	0.208	8.0	7.0	0.757	8.0	5.0	0.146	8.0	6.0	0.479
	2nd	8.0	6.0		8.0	6.0		8.0	6.0		8.0	6.0		5.0	6.0	
Meiboscale (grade)	1st	1.0	0.5	**0.020**	1.0	0.5	0.126	1.0	0.5	0.185	1.5	1.0	0.261	1.5	1.0	0.596
	2nd	1.5	1.0		1.5	1.0		1.5	1.0		1.5	1.5		1.5	1.0	
PB	1st	3.0	5.0	**0.004**	4.0	5.0	0.331	3.0	5.0	0.051	3.0	4.0	0.176	3.0	4.0	**0.029**
	2nd	2.0	4.0		3.0	5.0		2.0	3.0		2.0	3.0		1.0	3.0	
PB (%)	1st	62.5	60.8	**0.002**	62.5	61.6	**0.048**	60.0	62.6	**0.028**	63.1	58.5	0.890	50.0	60.1	0.110
	2nd	50.0	63.3		50.0	57.5		48.3	62.3		66.7	63.3		33.3	77.2	
TB	1st	6.0	6.0	0.901	7.0	7.0	0.389	6.0	6.0	0.905	6.0	5.0	0.459	6.0	6.0	0.506
	2nd	6.0	7.0		7.0	7.0		6.0	7.0		5.0	6.0		4.0	6.0	
Schirmer test (mm)	1st	4.0	5.0	**0.003**	4.0	5.0	**0.021**	4.0	5.0	0.296	4.0	4.0	0.651	4.0	5.0	**0.015**
	2nd	3.0	5.0		3.0	5.0		4.0	5.0		4.0	4.3		2.0	4.0	
FTBUT (sec)	1st	3.0	1.0	0.555	3.0	1.0	0.575	3.0	1.0	0.846	3.0	1.0	0.881	3.0	1.0	0.678
	2nd	3.0	1.0		3.0	2.0		3.0	1.0		3.0	1.0		2.0	1.0	
SPK (grade)	1st	0.0	0.0	0.187	0.0	0.0	**0.030**	0.0	0.0	0.815	0.0	1.0	0.647	0.0	0.0	0.672
	2nd	0.0	1.0		0.0	1.0		0.0	0.0		0.0	0.0		0.0	0.0	

DM: dermatochalasis; IQR: interquatile range; MGE: number of expressible meibomian glands; OSDI (A): subtotal of the frequency of symptoms; OSDI (B): subtotal of the frequency of activity limitation; OSDI (C): subtotal of the frequency of environmental factors triggering discomfort; LLT: average lipid-layer thickness; PB: number of partial blinks; B (%): partial blink rate; TB: total number of blinks; FTBUT: fluorescein tear-film break-up time; SPK: superficial punctate keratitis; *p*: statistical significance using the Friedman tes.

## Data Availability

The data presented in this study are available on request from the corresponding author.
